# The promises and challenges of early non‐small cell lung cancer detection: patient perceptions, low‐dose CT screening, bronchoscopy and biomarkers

**DOI:** 10.1002/1878-0261.12864

**Published:** 2020-12-14

**Authors:** Lukas Kalinke, Ricky Thakrar, Sam M. Janes

**Affiliations:** ^1^ Lungs for Living Research Centre University College London UK

**Keywords:** biomarkers, biomarkers, cancer, detection, screening

## Abstract

Lung cancer survival statistics are sobering with survival ranking among the poorest of all cancers despite the addition of targeted therapies and immunotherapies. However, improvements in tools for early detection hold promise. The Nederlands–Leuvens Longkanker Screenings Onderzoek (NELSON) trial recently corroborated the findings from the previous National Lung Screening Trial low‐dose Computerised Tomography (NLST) screening trial in reducing lung cancer mortality. Biomarker research and development is increasing at pace as the molecular life histories of lung cancers become further unravelled. Low‐dose CT screening (LDCT) is effective but targets only those at the highest risk and is burdensome on healthcare. An optimally designed CT screening programme at best will only detect a low proportion of overall lung cancers as only those at very high‐risk meet screening criteria. Biomarkers that help risk stratify suitable patients for LDCT screening, and those that assist in determining which LDCT detected nodules are likely to represent malignant disease are needed. Some biomarkers have been proposed as standalone lung cancer diagnosis tools. Bronchoscopy technology is improving, with better capacity to identify and obtain samples from early lung cancers. Clinicians need to be aware of each early lung cancer detection method’s inherent limitations. We anticipate that the future of early lung cancer diagnosis will involve a synergistic, multimodal approach, combining several early detection methods.

## Graphical abstract

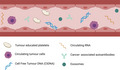

**Abbreviations**
AFB: Autofluorescence bronchoscopy
AUC: Area Under Curve
BROCK: BROCK University Lung Cancer Screening and Risk Prediction model
cfDNA: Circulating cell‐free DNA
COPD: Chronic obstructive airways disease
CT: Computerised Tomography
CTC: Circulating whole tumour cells
ctDNA: Circulating cell‐free tumour DNA
DNA: Deoxyribonucleic acid
EBUS: Endobronchial Ultrasound
EVs: Extracellular vesicles
HUNT: Nord‐Trøndelag Health Study
LDCT: Low‐dose Computerised Tomography
LSUT: Lung Screen Uptake Trial
LUSC: Squamous Cell Lung Cancer
MAYO: Solitary Pulmonary Nodule Malignancy Risk Score
MiLD: Multicentric Italian Lung Detection
miRNAs: MicroRNAs
NELSON: Nederlands–Leuvens Longkanker Screenings Onderzoek
NLST: National Lung Screening Trial
NSCLC: Nonsmall cell lung cancer
PLCOm2012: Prostate, Lung, Colorectal and Ovarian Cancer Screening Trial modified 2012
SABR: Stereotactic body radiotherapy
SES: Socioeconomic status
SNP: Single Nucleotide Polymorphisms
TEPs: Tumour educated platelets
TNM: Tumour Node Metastasis model
UK: United Kingdom
US: United States
VDT: Volume doubling time
VOCs: Volatile organic compounds

## 1 Introduction

Lung cancer is the leading cause of death from cancer worldwide. To improve survival, there needs to be a shift in the stage of disease at which it is first diagnosed along with timely and successful treatment. Lung cancer is broadly classified into small cell lung cancer and nonsmall cell lung cancer (NSCLC). The early detection of NSCLC, which accounts for ~ 85% of all lung cases [1], will be the focus of this review. NSCLC is further divided into its two main subtypes: adenocarcinoma, typically located in the lung peripheries, and squamous cell carcinoma typically found in the central airways. Ever‐increasing molecular subtyping is now used giving more precise treatment strategies and better prediction of clinical trajectories.

**Fig. 1:**
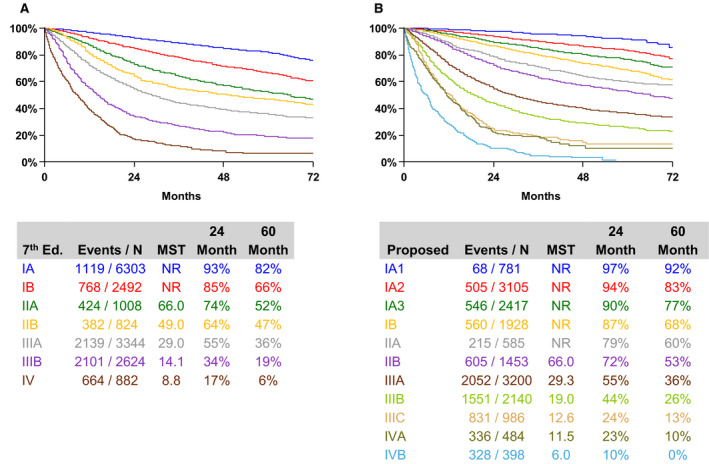
Overall survival by clinical stage according to the eighth edition of the TNM classification for Lung Cancer. Figure re‐published with permission from authors.

Sadly, three quarters of patients with lung cancer are diagnosed at stage III or IV [2], when disease has spread to lymph nodes or other organs and is incurable. Earlier detection at stages I and II means better survival as radical (potentially curative) treatment approaches can be employed; 92% of those patients diagnosed with stage IA1 disease (tumour < 1 cm and no involvement of main bronchi or nodal disease) survive 5 years or more, compared to just 10% for patients diagnosed with stage IV disease (cancer in both lungs, in the lungs’ lining or spread to another organ) []. Importantly, even a small increase in tumour size from < 1 cm (stage IA1) to > 2 cm (stage IA3), reduces 5‐year survival to 77%. As soon as local nodes become involved or the tumour is larger than 5 cm or it invades local structures (Stage IIB), 5‐year survival drops to 53% (Fig. 1).

Five‐year survival rates have historically been worse in the United Kingdom (UK) compared to comparator countries worldwide [4]. Causes of this discrepancy are thought to be a combination of wide geographical variation in access to curative surgical resection or radical radiotherapy [5], and late or delayed diagnosis, with delays occurring in presentation, primary care and secondary care. Patients in England are less likely to have their NSCLC diagnosed at stage I compared to those in the United States (US) (15% vs 24%), and this results in avoidable deaths [6]. This study concludes that in the UK there are 98 excess deaths per one thousand patients with histology‐proven NSCLC.

Although details of specific treatments are outside of the remit of this review, early detection and diagnosis and definitive treatment are intimately related to lead time bias, especially when considering screening populations for lung cancer, a topic discussed shortly. There also needs to be equity in treatment access [7, 8]. Lead time bias occurs when a diagnostic approach merely identifies the disease earlier and gives the impression that survival is prolonged when in fact the treatment has no effect on outcome [9]. Hence, the requirement for an effective treatment if one pursues earlier diagnosis. The median volume doubling time (VDT) of NSCLC is believed to be 121 days with 41% of lung cancers having a VDT of < 100 days [10], corroborating the need to act quickly Yang *et al*. sought to correlate the timing of lobectomy with survival outcome in just under 5000 patients who had stage IA squamous cell lung cancer (LUSC) between 2006 and 2011. They found that a delay of more than 37 days from time of diagnosis to surgery was associated with worse survival [11]. The overall 5‐year survival was 58%, the study using the older Tumour Node Metastasis (TNM) staging system without further subtyping of stage IA into IA1, IA2 and IA3.

The UK Lung Cancer Coalition ‘25 by 25’ [12] builds on the NHS's Long Term Plan to diagnose 75% of cancers at an early stage. The Coalition aim to improve lung cancer's 5‐year survival rate to 25% by 2025 [13]. The current UK 5‐year survival (unspecified stage) has been modestly increasing and currently sits at 16% [14], having previously been 9% in 2005 [12]. The National Lung Cancer Audit has driven improvements by systemically reviewing nationwide data on lung cancer outcomes in the UK. The most recent data show there has been a 5.4% increase in the number of lung cancer operations and importantly less variation between trusts [15]. There has also been progress made in treatment; stereotactic body radiotherapy (SABR) is now used to treat patients unfit to undergo surgical management [8, 16, 17], and targeted therapies and immunotherapy are now available, but these are licensed only in advanced disease and offer modest benefits in the realm of months [18, 19]. They are also costly.

This review starts by describing strategies that have aimed to change patient behaviour towards lung cancer diagnosis, followed by a review of biomarkers proposed as standalone lung cancer early detection tools. The review then examines low‐dose computerised tomography (CT) screening and the adjunct use of biomarkers. It finishes by exploring the early detection and treatment of central lung cancers.

## 2 What are the causes of late detection?

Many individuals with lung cancer have symptoms for several months before they seek medical help. One modifiable trait is the consulting behaviour of patients at risk of having lung cancer. A UK study found the mean delay between symptom onset and date of diagnosis was 12 months, a figure that persisted when accounting for patients with operable, early disease [20]. This was supported by a Swedish study [21] and challenges the belief that lung cancer is asymptomatic until advanced. One Scottish study separated symptoms into participant‐defined and health professional‐defined, the latter being based on a checklist of common symptoms [22]. Here, the median time from participant‐defined first symptoms to consultation with a healthcare professional was 21 days. However, most participants, even those having described themselves as having none, reported additional symptoms when probed by the clinician using the symptom checklist, suggesting that these symptoms had been ignored/deemed not important. The median time from earliest reported checklist symptoms until consultation was 99 days. This nearly matches the aforementioned median VDT [10]. The delay in presentation of patients' to a healthcare professional has been attributed to patients displaying ‘medical nihilism’, believing lung cancer to be incurable; fear; lack of interpretation that their symptoms are serious [23] and stigma around smoking [24]. It has been shown that current smokers are more likely to experience respiratory symptoms but less likely to consult about them [25].

Behavioural intervention can promote earlier presentation to healthcare professionals amongst those at risk of lung cancer. The rewards are being reaped from an initiative that was started in 2011 in Leeds [26]. Here, a two‐pronged approach to introduce a stage shift through improving symptom awareness (to both patients and health care professionals) was rolled out. Firstly, a primary healthcare educational package was delivered highlighting what the local chest X‐ray referral guidelines were. Secondly, a marketing communication campaign was developed with the tagline: ‘Got a cough, get a check’. There was a subsequent 80% increase in chest X‐ray referrals and importantly, an 8.8% increase in the proportion of patients diagnosed with stage I/II lung cancer.

## 3 Can molecular biomarkers be used as a standalone test for early lung cancer detection?

The advances in multi‐omic technologies have informed us about the molecular biology underpinning lung carcinogenesis leading to an explosion in biomarker research. The vast intertumour heterogeneity that exists makes identifying a ‘one size fits all’ (maximally sensitive) biomarker to identify all lung cancer cases difficult. Rarer mutations may not be accounted for. The common mutations in lung cancer genes are also present in ex‐smokers in histologically ‘normal’ tissue [27], accumulate with age [28] and in chronic lung conditions such as lung fibrosis [29] and chronic obstructive pulmonary disease (COPD) [30], limiting the specificity of many candidate biomarkers. Also, this elusive ideal biomarker should ideally be involved in early carcinogenesis to limit lead time bias.

**Fig. 2:**
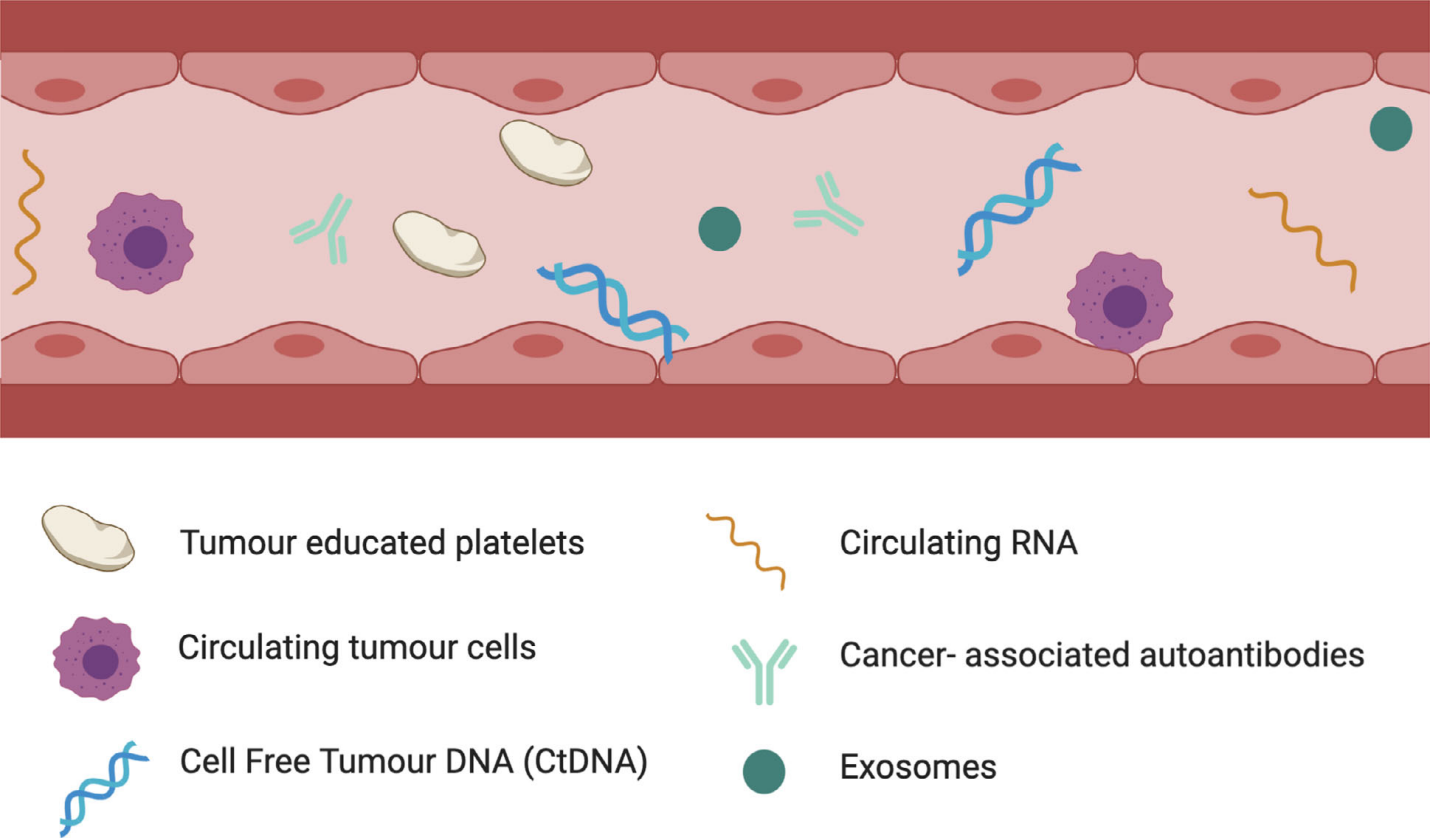
Image depicting all tumour‐derived components that can be detected in blood. Produced using BioRender.

There are many tumour‐derived components that can be detected in blood: cancer‐associated auto‐antibodies, circulating cell‐free tumour DNA (ctDNA), circulating cell‐free tumour RNA – the most abundant type being microRNAs (miRNAs), circulating whole tumour cells (CTC), tumour educated platelets (TEPs) and exosomes. Figure 2 depicts these components.

### 3.1 Cancer auto‐antibodies

The EarlyCDT‐Lung trial has shown promise in increasing the pretest probability of lung cancer in a high‐risk population [31]. This study involved a blood test that measures auto‐antibodies to the lung cancer‐associated antigens p53, NY‐ESO‐1, CAGE, GBU4‐5, Annexin 1 and SOX2. More recently, Hu‐D was added to the panel. In contrast to detecting cancer‐associated antigens as markers of tumour burden, detection of auto‐antibodies may theoretically be better suited to screen for early disease, as there is an early amplification signal in auto‐antibody levels produced during the immune sensing phase [32]. Initial clinical validation of the six‐auto‐antibody panel notably showed no significant differences in detection efficacy among the various stages of disease, demonstrating potential utility in early disease. Meta‐analysis of four studies explored the diagnostic value of the 7‐panel assay – shown to be superior to the 6‐panel assay – and showed a pooled sensitivity of 47% (range 37–66%), specificity of 90% (range 84–91%) and area under curve (AUC) of 0.90 (range 0.87 and 0.93) but of note, the *P* value was 0.00 indicating heterogeneity between studies [33]. A sensitivity of 47% suggests it is not a useful standalone lung cancer rule in test, but, as discussed later, this auto‐antibody test may have utility when combined with other tests.

### 3.2 Circulating cell‐free tumour DNA

Detecting circulating ctDNA has been applied successfully for the monitoring of patients with existing tumours, in particular tracking drug‐resistant EGFR mutations [34]. It has not been useful for early diagnosis of cancer because of both low sensitivities in detecting small tumours that only shed minute quantities of DNA into the blood but also due to a lack of knowledge of the primary tumour to guide mutational analysis. The ctDNA fraction (equivalent to mutant allele fraction) in early disease is low, representing ~ 0.01–1% of total circulating cell‐free DNA (cfDNA) [35]. A 1 mL of blood sample contains an amount of DNA equivalent to the genomes of 2000 cells, so one can see the limits in detecting such mutant variants. Diamandis *et al*. [36] have shown that when a tumour diameter drops below 10 mm, not a single mutant DNA copy is likely to be retrieved in 10 mL of plasma. Furthermore, a 1‐mm tumour is likely to be associated with just one copy of ctDNA in the entire circulation. These calculations were built from work using circulating fetal DNA in the maternal circulation. Assuming the fetus is a ‘foreign body’ (used as a proxy for tumour presence) and the concentration of fetal‐derived DNA correlates with fetal weight; if the rate of diffusion of DNA into the circulation is equal between normal and malignant tissue, then a 10‐cm^3^ tumour correlates to 0.1% of ctDNA in the circulation. These assumptions have some limitations. The rate of diffusion of ctDNA is unlikely to match that of normal tissue and different tumours are likely to shed DNA at different rates. This model does however serve to illustrate the limitations of ctDNA in early cancer detection and explains why it is currently clinically utilised in tracking resistance and relapse when tumours are larger and/or more aggressive.

Studies using library sequencing adaptors for genes selected at a population level have so far shown sensitivities of 50% for patients with stage I NSCLC [37] and 59% for patients with stage I or II NSCLC [38]. This approach reduces the test's specificity as some of these mutated genes can also be present in noncancerous tissue. A recently published machine learning method was able to distinguish somatic clonal haematopoiesis mutations from tumour‐derived mutations based on fragment size and mutational signature. The authors were able to detect ctDNA from 42% of patients with stage I lung cancer [39]. Another paper used cell‐free DNA fragmentation length and position within the genome to diagnose cancer [40]. The team discovered that the fragments of tumour‐derived ctDNA varied more in length compared to cfDNA, being typically shorter by about 3–6 bases. The sensitivity of another machine learning model based on fragmentation features for detecting lung cancer was 100% (of various lung cancer stages), and 73% when used in patients with stage I Iung cancer. These fragmentation models indicate that instead of seeking to detect single variants, akin to finding needles in haystacks, a more superficial approach such as analysing fragment size may be required for detection of early‐stage disease.

Another approach is to analyse the methylome of ctDNA. Akin to analysing fragment length, information can be derived from a nonspecific portion of genomic DNA regions rather than searching for individual mutations as there are global changes in DNA methylation during the initiation and progression of tumourigenesis [41], that are also tissue‐ and cancer‐type specific [42]. Chen *et al*. [43] recently showed that PanSeer, a noninvasive blood test detecting methylation aberrations in ctDNA, detects cancer in 95% (95% CI: 89–98) of asymptomatic individuals who were diagnosed up to 4 years after the test. Lung cancer patients were included in the dataset, but the specific sensitivities for lung cancer are not quoted.

The healthcare biotechnology company GRAIL Inc aim to produce a multicancer early detection test that identifies abnormally methylated ctDNA [44]. GRAIL investigators showed that whole‐genome bisulfite sequencing outperformed whole‐genome and targeted sequencing approaches for multicancer detection across many cancer stages at high specificity [45]. The data on lung cancer specifically have not been published.

### 3.3 MicroRNAs

MicroRNAs are short noncoding, stable RNA sequences that regulate gene expression post‐transcriptionally. Tumour‐secreted miRNAs are detectable in the circulating blood [46]. Studies evaluating miRNAs as an early detection tool are discrepant, potentially due to issues around sample sizes, processing techniques and lung cancer pathology. A meta‐analysis of 28 studies showed a pooled sensitivity and specificity of miRNA as biomarkers of 0.75 and 0.79, respectively [47]. Subgroup analyses show that miRNAs are more effective in detecting NSCLC in Caucasian populations compared to Asian populations; panels of miRNAs were superior to individual, as were blood‐derived miRNAs compared to sputum‐derived miRNAs. The largest study in this meta‐analysis included only 320 subjects. Future studies will perhaps focus on specific histological subtypes of cancer as miRNAs vary and reflect lung cancer subtypes [48].

### 3.4 Circulating tumour cells

Migration of tumour cells into the blood stream is an early event that occurs during carcinogenesis, resulting in CTCs. CTCs have been found to be present in as many as 80% of patients with stage I/II NSCLC [49]. CTCs are, however, rare and both difficult and costly to isolate, so cementing a role for them in clinical practice appears challenging [50]. A recently published multicentre, prospective cohort study sought to assess whether CTCs can be used as a standalone lung cancer screening tool amongst high‐risk patients [fulfilling National Lung Screening Trial (NLST) criteria] with COPD [51]. The study included three annual LDCT visits to determine the sensitivity of CTCs; the primary endpoint was the diagnostic performance of CTCs at the first screening visit. Out of 614 patients who attended the first visit, 27 patients had CTCs detected (seven patients had malignant looking CTCs that had to have all four cytological features of malignancy and 20 patient had CTCs with ‘uncertain’ malignant features, where the cells only had to have one cytological feature). There were 19 lung cancers detected (screen detected at T0 and confirmed on histology); of which five had CTC detected (three malignant, two uncertain malignant features) giving a sensitivity of 23.8%, too low to justify use of CTC isolation as a standalone tool. This study used a single method of CTC extraction, and other extraction methods may increase CTC yield. Of interest, the authors found the overall risk of developing lung cancer was almost three times higher than that in NLST and Nederlands–Leuvens Longkanker Screenings Onderzoek (NELSON), supporting COPD as being an independent risk factor for lung cancer development [52].

It seems the sensitivity of detection of CTCs challenges their use as a screening tool but improved methodology may help. Meanwhile, their use as a prognostic marker in more advanced disease looks more exciting [53]. For instance, pulmonary vein CTCs collected at surgery revealed higher mutation overlap with future metastasis than with the primary tumour, suggesting a causal role in disease relapse [54]. Their presence may therefore guide relapse treatment strategies.

### 3.5 Tumour‐educated platelets

Platelets are known to interact with tumour cells and can affect their growth and invasive capabilities [55]. Platelets are receptive to growth factor release from tumour cells that cause specific spliced RNA profiles within platelets that can be sequenced [56]. Such analysis was able to discriminate 53 locally advanced NSCLC patients from 377 healthy individuals with an AUC of 0.89 [57]. The definition of locally advanced was not given. A key challenge with TEPs appears to be the transcriptome is exquisitely sensitive to external conditions so future prospective studies must ensure the patient and control samples are collected under the same conditions.

### 3.6 Extracellular vesicles

Extracellular vesicles (EVs) are cell‐derived structures that are present in plasma. They traffic biological material across membranes to maintain compartmentalisation of molecules, but also serve as a cross‐talk communication system that can influence tumour related pathways within the tumour microenvironment [58]. Exosomal RNA is more resistant to RNAse activity than free form circulating RNA [59] and is more sensitive in detecting epidermal growth factor mutations compared to matched ctDNA [60]. Proteins are also carried by extracellular vesicles; Vykoukal *et al*. [61] used mass spectrometry to identify over 600 proteins, four of these were able to distinguish adenocarcinoma (stages I and II) from healthy controls with an AUC of 0.90. Despite EVs being a trove of tumour cell‐specific information more resistant to degradation and hence very appealing, a limiting factor in clinical practice will be their challenging isolation from plasma.

### 3.7 Volatile organic compounds

Alterations in key metabolic pathways can be detected in exhaled breath and are strongly linked to the transformation of healthy cells to malignant cells [62]. A breath test is noninvasive and cheap to perform. Exhaled breath analysis detects the concentrations of volatile organic compounds (VOCs), which reflect underlying pathophysiological processes [63]. Patterns of VOCs have been shown to alter when lung cancer is present [64, 65, 66, 67, 68, 69, 70, 71]. A recent study demonstrated that exhaled breath analysis could differentiate subjects with lung cancer from healthy individuals with a sensitivity of 94%, a negative predictive value of 85% and an AUC of 0.76, although 75% of patients had disease at stage III or later stages [72]. Specificity is an issue, as VOCs are also present in healthy individuals, and hence, there is no consensus on what constitutes a normal reference value. Antoniou *et al*. provide some insight as to why there are no validated, approved breath tests despite their strong biological basis: studies have small numbers, many of which are not multisite so reproducibility is not ascertained; breath collection and analysis methods need to be standardised; findings have not been prospectively validated and development requires fruitful collaborations between academia and industry [71].

### 3.8 Bacterial biomarkers

The mucosal surface in the lung is colonised by a diverse bacterial community. The interplay between the host response to microbes and lung carcinogenesis is of increasing interest. The development of lung cancer is associated with chronic inflammation [73] but how the tumour exploits the local immune system to create a permissive environment for growth is not well understood. The microbiota is believed to orchestrate the balance between tumour promoting inflammation and tumour immunity. Interestingly, a recent study showed that germ‐free or antibiotic treated mice were protected from lung cancer development, despite having *KRAS* mutations and *p53* loss [74]. Commensal bacteria were shown to activate γδ T cells that provoked the inflammatory response necessary for carcinogenesis. It has also been shown patients with lung cancer carry a different and less diverse microbiota compared to healthy controls [75].

The microbiome of saliva has shown promise as a biomarker in a small study of 61 patients with lung cancer and 25 controls. Specifically, detection of both *Capnocytophaga* and *Veillonella* species has a 84% sensitivity and 86% specificity in distinguishing patients with lung squamous cell carcinoma (all with stage II disease or above) from control subjects [76]. The results were less convincing for adenocarcinoma.

We foresee a definite role for biomarkers as an adjunct tool to improve pretest probabilities for screening, assist our decision making in nodule management and help guide decisions in early central cancers. Finding a standalone, highly sensitive biomarker for early detection that is subsequently validated in a highly statistically powered, multicentre study to ensure reproducibility however has significant cost and time challenges to commercialisation.

## 4 Early detection of parenchymal lung cancers: low‐dose CT screening and integration with biomarkers

Earlier attempts to screen for lung cancer using X‐ray and sputum testing failed to show a mortality reduction [77], the prime objective of a screening programme [78]. The success of two trials showing reduction in mortality using spiral low‐dose CT screening has catapulted lung cancer screening onto the agendas of health policy makers worldwide due to the speed of image acquisition, low radiation dose and high sensitivity. To understand where biomarkers show promise, one needs to be aware of the limitations of LDCT screening.

Two landmark studies – NSLT [79] and NELSON [80] – have demonstrated that LDCT screening reduces lung cancer mortality. LDCT screening does however have challenges that need solutions, or more likely, compromises. The trial methodologies and results are summarised in Table 1. As compared to the NSLT trial, the NELSON trial included younger patients with less smoking exposure; fewer females; different screening intervals; and the comparison group were not screened. Importantly, NELSON also used a volume‐based nodule‐management protocol rather than the diameter‐based protocol of NLST; positivity depended on initial lesion volume and in later screening rounds, VDT. This resulted in a much lower false‐positive rate of 1.2%. To put into perspective the promise of LDCT screening, the number of women needed to screen to prevent one death from breast cancer is 781 [81]; compare this to the NLST trial which needed to screen 320 patients to prevent one death from lung cancer [79].

**Table 1:** Overview of findings from NLST and NELSON LDCT screening trials.

	National Lung Screening Trial (NLST)	Nederlands–Leuvens Longkanker Screenings Onderzoek (NELSON)
Trial design	RCT of annual LDCT or chest X‐ray Diameter‐based protocol for lesion measurement	RCT of 4 CT scans over 6 years (rounds 1/2/3/4: baseline, year 1/3/5.5) vs no screening Volume‐based protocol for lesion measurement
Number of participants (male/female)	53 454 (31 532/21 922)	15 792* (13 195/2594) * 3 unknown sex
Inclusion criteria	Aged 55–74 > 30 pack year history Current smoker or quit < 15 years	Aged 50–74 Current or former smokers (< 10 years since quitting) who had smoked > 15 cigarettes a day for > 25 years or > 10 cigarettes a day for > 30 years
Key results	20% relative risk reduction in lung cancer mortality 7% reduction in overall mortality False‐positive rate: 96.4% 63% cancers detected stage 1	24% relative risk reduction in male lung cancer mortality 33% relative risk reduction in female lung cancer mortality (not statistically significant due to small sample size) All‐cause mortality rate ratio 1.01 False‐positive rate: 1.2% 71% cancers detected stage 1

The actual number of lung cancer lives saved using the NLST inclusion criteria was shown to only prevent 10% of annual lung cancer deaths in the US [82]. The U.S. Preventive Services Task Force has since increased the age cut off for their current screening service to 80 years of age [80]. NLST criteria are insensitive for detecting stage I/II cancers: in one large US cancer centre only 48% of stage I/II cancers would have met NLST inclusion criteria for screening [83]. Using model‐estimated risk predictors is superior to just using smoking and age [84]; numerous such prediction models now exist (e.g. Liverpool Lung Project [85], Prostate, Lung, Colorectal and Ovarian Cancer Screening Trial (PLCOm2012) [86], Nord‐Trøndelag Health Study (HUNT) [87]). There have been several smaller but successful LDCT screening trials in the UK [88, 89, 90]. In February last year, the UK NHS announced the targeted Lung Health Check Programme, rolling out LDCT screening across 14 sites in England [91], a likely stepping stone before it is implemented nationwide.

The incidence of adenocarcinoma has risen in the US since 2006 [92] and has now surpassed lung squamous cell carcinoma [93]. Lung adenocarcinoma is often located at the lung periphery and can display a wide variety of microscopic features [94], and these observations necessitate a revised classification system [95]. The incidence in men has stabilised but is rising in women [96], and this cannot be explained by smoking behaviour alone [97]. The declining rates of smoking may underlie the rise in relative proportion of lung cancers in never‐smokers. In the UK, it is estimated that nearly 6000 people who have never smoked die of lung cancer every year [98]. These patients are more commonly female and harbour an oncogene‐driven adenocarcinoma [99]. Current LDCT screening selection criteria will miss this important population, in addition to those patients that have a lower smoking history than what eligibility criteria demand. It is estimated that < 50% of incident lung cancer cases are amongst individuals who are eligible for screening [100]. Therefore, early detection methods are desperately needed that allow us to screen a wider population, potentially then leading to more intensive screening such as LDCT.

Molecular biomarkers could routinely aid LDCT in three ways: by selecting patients who will gain the most benefit (most deaths being averted while being fit enough to proceed with treatment), using the least resources (for economic and radiation reasons) while potentially including people that would not qualify for CT screening through current criteria; assisting in interpreting nodules and supporting decisions on interval screening schedules.

## 5 Molecular biomarkers for selecting patients for CT screening, aiding nodule interpretation and to determine CT scanning intervals

Early detection is only worthwhile if effective treatment can be offered. Screening highly co‐morbid patients who are unlikely to be offered treatment leads to lead time bias. The criteria determining who is ‘fit’ to receive curative treatment are broadening with the advent of SABR [8, 16, 17].

### 5.1 Patient selection

Young *et al*. have compared the utility of a gene‐based model [germline single nucleotide polymorphisms (SNP)] with the PLCOm2012 risk model in predicting risk of developing lung cancer. While the gene‐based risk model has comparable predictive utility as the PLCOm2012 risk model (unpublished data, personal communication with author), it may be more efficient at identifying who will benefit most from lung cancer screening [101]. This gene‐based risk model combines the genotypes of 12 SNPs with a simplified clinical score for lung cancer validated in a subgroup of the NLST (*N* = 10 054 subjects). Young *et al*. found that when NLST participants that had been initially classified according to the PLCOm2012 risk model were reclassified according to the personalised gene‐based risk model, 41% of them changed risk tertile (unpublished data, personal communication with author). The authors believe the SNP data reflect tumour aggressiveness [102] and re‐classifies some of the PLCOm2012 intermediate risk individuals into the high‐risk tertile (where net screening benefit decreases due to competing causes of death, more aggressive cancer and decreased treatment tolerability). Similarly, patients in the PLCOm2012 high‐risk tertile with less aggressive tumour biology are re‐assigned into the intermediate risk tertile where screening benefits are greatest. When stratifying by risk quintiles (*N* = 5), the number of individuals needed to screen to prevent one lung cancer across quintiles 2–4 (intermediate risk) was reduced from 182 with the PLCOm2012 model to 98 using the gene‐based approach – twofold more efficient [103].

A panel of five proteins was shown to outperform the current US screening eligibility criteria in predicting lung cancer in a cohort of 63 patients with lung cancer and 90 matched controls (sensitivities 0.63 vs 0.42). It was also more specific (0.95 vs 0.86), again providing evidence that biomarker risk profiling may aid patient selection to screening programmes [104].

A group in Scotland used the seven‐auto‐antibody panel previously described to select adult patients (50–75, > 20 pack years, living in the most deprived quintile of deprivation) for LDCT screening. Participants were randomised to either undergo the Early CDT‐Lung test (and if positive, LDCT scanning 6 monthly for 2 years; if negative, to receive standard clinical care) vs no Early CDT‐Lung test and standard clinical care in the control arm. Their aim was to see whether this intervention reduces the rate of late‐stage cancer diagnoses [105, 106]. At 2 years, there were 127 lung cancers detected from 12 208 participants randomised. In the intervention arm, 33/56 (58.9%) lung cancers were diagnosed at stage III/IV compared to 52/71 (73.2%) in the control arm. The hazard ratio for stage III/IV presentation was 0.64 (95% CI 0.41, 0.99). There were nonsignificant differences in lung cancer and all‐cause mortality after 2 years. Full data from this study are awaited and detail on whether the survival advantage can be attributed to the blood test or simply the increased CT rate in the active arm. Given the surprisingly low incidence of lung cancer, the absolute risk reduction in late‐stage diagnosis in the intervention arm was only 0.3%. This low lung cancer incidence could be attributed to the fact that the Early CDT‐Lung test misses early lung cancers that may have been detected if all participants had received LDCT.

### 5.2 Nodule interpretation

Molecular biomarkers may help stratify low‐ and high‐risk nodules, giving clinicians more confidence when interpreting scans. False‐positive test results following screening are a problem, they cause psychological morbidity [107] and rarely death; there were six deaths within 60 days of an invasive procedure in the NLST amongst patients who had false‐positive scan results [79]. Indeterminate scans are also common, and these require further screening resulting in increased radiation exposure for recipients. In NELSON, 20% of scans within the first screening round were indeterminate [80]. In the UK, clinicians typically use the BROCK University Lung Cancer Screening and Risk Prediction model (BROCK) to aid solid nodule risk prediction [108, 109] and the British Thoracic Society Nodule guidelines to determine interval scanning. The problem with these models is there is interobserver variability in measuring diameters, volumes and other factors used in the models, however the use of computer aided detection should reduce this [110]. Of note, the BROCK model has utility in solid nodules, but not for subsolid nodules (ground glass and part‐solid nodules).

A protein‐based model that combines clinical risk factors with 13 proteins to classify indeterminate lung nodules – those deemed to have a clinician assessed pretest probability of malignancy of < 50% – has been proposed by Silvestri *et al*. [111]. In a prospective, multicentre observational trial with 685 patients, subgroup analysis of 178 patients who had one nodule of between 8 and 30 mm diameter showed that use of the 13‐protein classifier outperformed other validated risk prediction models and positron emission tomography scans (*P* < 0.001) and demonstrated a sensitivity of 97% and a negative predictive value of 98% in distinguishing benign from malignant nodules. Malignancy was confirmed histologically; benign disease was confirmed either by histology or radiologically (nodule size remained stable or reduced).

The addition of the 7‐auto‐antibody panel has shown to also distinguish between benign and malignant nodules [112]. It increased the relative risk of malignancy by 2.7 for nodules measuring 4–20 mm. Lowering the risk threshold to include more participants (using the Solitary Pulmonary Nodule Malignancy Risk Score (MAYO) [113]) and adding this antibody panel increased sensitivity by reclassifying a proportion of patients who had been wrongly classified as negative by MAYO criteria. Another group more recently used a combination of four other auto‐antibodies that could detect malignant nodules with an AUC of 0.78 in a subgroup of indeterminate nodules (8–20 mm) amongst an independent validation cohort of 250 patients [114]. These results, as with all novel biomarkers, need validation in much larger studies.

Philips *et al*. [115] reanalysed the dataset of around 300 patients who donated breath for an earlier LDCT study [116]. Accordingly, they identified a VOC signature, termed Mass Abnormalities in Gaseous Ions with Imaging Correlates (MAGIIC), that was able to predict which pulmonary nodules were malignant with an AUC of 0.88 [115]. These results remain to be validated in a larger study.

The plasma of 60 healthy controls and 150 patients who underwent surgery for suspicious nodules was collected and tested for [117] methylation at the promoters of five genes known to be differentially methylated in the circulating DNA of patients with NSCLC (SOX17, TAC1, HOXA7, CDO1 and ZFP42) [118, 119]. A three‐gene combination of the best individual genes had a sensitivity and specificity of 93% and 62%, respectively, and an AUC of 0.77 for identifying malignant nodules.

Multicentric Italian Lung Detection trial (MiLD), a randomised LDCT trial in Italy (annual or biennial LDCT vs observation only), collected plasma prospectively and then retrospectively analysed for a 24‐miRNA classifier [120] previously tested for the prediction of both the risk of lung cancer development and presence of aggressive disease [121]. The classifier grouped patients into tertiles of high, intermediate or low risk of having lung cancer. For patients who had suspicious nodules detected and classified as being high risk by the miRNA classifier, the false‐positive rate was reduced from 19.4% (LDCT screening alone) to just 3.7%. For all patients across both arms, the classifier had a negative predictive value of 99% indicating the miRNA panel may also have a use as a pre‐LDCT test to select patients for screening. Other smaller studies have supported the use of miRNA in distinguishing malignant and benign nodules [122, 123].

### 5.3 CT screening intervals

Blood miRNA isolated from the MiLD trial has also shown promise in selecting the timing of the next LDCT interval scan [124]. Those with a negative miRNA and initial negative CT had a 3‐year follow‐up scan. There were no detrimental effects on stage 1 resection or interval cancer incidence in the 3‐year double‐negative group suggesting the use of MiRNA can provide reassurance when scheduling interval scans and can limit unnecessary radiation exposure.

## 6 Future use of molecular biomarkers in lung cancer screening: nonsmokers, aggressive tumours and stopping screening

First, biomarkers are needed to detect lung cancer in those that do not qualify for lung screening – light or never‐smokers [125]. Second, from the NELSON dataset, at year 3 (round 3), 3.9% of all tumours were stage IV, whereas 2.5 years later, 13.1% of tumours were stage IV [126]. A biomarker that could identify individuals who develop these aggressive tumours at an earlier time point is much needed to avoid having to increase the frequency of scanning across the whole screened population. Finally, there is also a vacancy for a biomarker to help determine whether it is safe to stop screening. Currently in the US, screening stops once patients turn 81 or if it has been more than 15 years since they stopped smoking. So, if a 55‐year‐old quits smoking on starting screening, then screening will stop 15 years later, at which point their risk of lung cancer is one and a half times the risk of when they started screening [86, 127].

## 7 Challenges of LDCT screening

### 7.1 Uptake & adherence

The large LDCT trials have enrolled participants of a higher socioeconomic status (SES). Those from a lower SES, who are at highest risk of developing lung cancer, are less likely to engage with an offer of screening or persist with the whole programme [128]. Services need to be designed to reduce this participation bias. The Manchester Lung Health Check community‐based project is one such example; most participants were from the lowest decile of deprivation in England. Ever smokers aged between 55 and 74 were invited for a lung health check in mobile vehicles next to local shopping centres, with immediate access to LDCT for those at high risk (6‐year risk ≥ 1.51%, PLCOM2012 calculator) [90]. The Lung Screen Uptake Trial (LSUT) in London, aimed to increase enrolment to a LDCT programme using a postal invitation strategy for high‐risk patients to have a ‘lung health check’ [88] and tested invitational materials designed to reduce anxiety and increase uptake in people from lower SES. Patients in Islington and Hackney were randomised to either receive a more ‘traditional’ text heavy leaflet or one that was less text heavy and pictorially targeted some known psychological barriers to attendance. Sixty per cent of patients (across both arms) were from the most deprived Index of Multiple Deprivation quintile. Across both trial arms, uptake was higher than has ever been observed previously at 53% of those invited. The low‐burden intervention did not improve uptake overall but was the more equitable, better engaging those living in areas of highest deprivation and lung cancer incidence.

### 7.2 Incidental findings

More sophisticated imaging techniques means more incidental findings are detected [129].

The prevalence of incidental findings detected during the early phase of the NELSON trial was reported to be as high as 73%, but when these cases were dissected, those requiring further evaluation was only 7% [130]. In the baseline round of NLST, 10% of subjects had a negative (for lung cancer) screen but other findings warranting further evaluation [79]. Incidental findings cause anxiety [131] and are also costly. In the US, 46% of the money reimbursed for screening from Medicare was for incidental findings [132]. Incidental findings, however, may offer an opportunity to discuss primary prevention measures. Sixty‐one per cent of patients that took part in the London LSUT trial had coronary artery calcification. Ninety‐eight per cent of patients had a QRISK score of > 10%, who would qualify for a statin as primary prevention against cardiovascular disease; however, only 56% of patient reported statin use [133].

### 7.3 Oncogenic risks of radiation dose

With a LDCT scan, patients are exposed to an average dose of 1.5 mSv of radiation [134], approximately half of the 2.7 mSv natural ambient yearly exposure in the UK [135]. It is estimated that the ratio of LDCT‐caused cancers to lung cancer deaths averted by LDCT is 1:20 [136]. A biomarker to help determine appropriate screening intervals would likely shift the benefit‐harm ratio even more favourably.

### 7.4 Resource demands of LDCT screening

In order to deliver LDCT screening nationally, there needs to be ample radiographers, radiologists and CT scanners. There is currently a national shortage of radiologists (of over 1000 consultants) to deliver the current workflow of the NHS, even without LDCT scanning [135]. There are also shortages in CT scanners: the latest figures are from 2014, where in the UK there were 9 CT scanners per million population, compared to 35 in Germany [135]. Artificial intelligence deep learning algorithms are being developed to detect lung cancer, which may assist with workflow [137, 138].

### 7.5 Tissue diagnosis

To comprehensively genotype tumours requires an adequate quantity and purity of tissue [139]. Most early cancers are detected in the lung parenchyma. Sixty per cent of nodules are in the outer third of lung [140] and can be challenging to sample as nodules move with respiration [141]. Current methods for tissue acquisition include percutaneous CT guided lung biopsy and bronchoscopic trans‐bronchial biopsy. Percutaneous CT biopsy can be very accurate with a diagnostic sensitivity of 85.7–97.4% [142].

Navigational bronchoscopic technology has been increasingly used in sampling lung nodules. Rendering of CT imaging to create a virtual bronchoscopic path to nodules can help guide the bronchoscopist. The addition of fluoroscopy and radial endobronchial ultrasound (EBUS) can then identify when the nodule has been reached for sampling but not visible [143]. Electromagnetic navigation has most commonly been adopted. This is a system that guides a metal catheter tip to the tumour using a virtual map [144]. Cone beam CT has been used to allow almost real time biopsies as imaging is performed just prior to biopsy and can be combined with novel navigational methods. These bronchoscopic techniques have sensitivities in the range of 60–70% [145, 146], but use of robotic bronchoscopy may change this, housing a flexible and steerable bronchoscope and catheter [147]. The sensitivities for robotic bronchoscopy ranged from 69% to 77% in 165 patients treated across four different centres [148].

### 7.6 Overdiagnosis

Overdiagnosis refers to identifying and potentially treating problems that were never going to cause harm [149], the patient will die from something other than their lung cancer. Overdiagnosis was felt to represent 18% of all lung cancers detected in the NLST trial [150] but with an additional median 6 year of follow up data, overdiagnosis is reduced to 3% [151]. In a similar vein, it must be noted that although lung cancer mortality is reduced by LDCT screening, NELSON failed to show a reduction in all‐cause mortality [79, 80]. Lung cancer screening is not unique with this discordance being seen in most cancer screening trials [152, 153]. Proposed reasons for this include harms due to interventions resulting from screening; biases in interpreting causes of death and small sample sizes. Biomarkers may improve overdiagnosis by detecting more aggressive tumours or enriching the screened population with those individuals most likely to benefit. This may favourably alter the all‐cause mortality rates.

## 8 Early detection of central airway cancers

Bronchoscopic detection and surveillance has allowed pre‐invasive lesions to be identified and followed closely [154, 155, 156, 157]. Importantly, tissue collected longitudinally for analysis provides a window to unravel the dynamic molecular events that occur in early lung squamous cell cancer (LUSC) development. Before progression to invasive LUSC, there is a step‐wise evolution of ever more disordered pre‐invasive lesions, ranging from mild and moderate dysplasia (low‐grade lesions) to severe dysplasia and carcinoma‐in‐situ (high‐grade lesions) [158]. High‐grade lesions are more likely to progress to invasive cancer than low‐grade lesions [156, 159, 160, 161, 162, 163], with progression rates varying between 43% and 87% across different studies that have different endpoints [155, 161, 164, 165, 166]. The prevalence of severe dysplasia and carcinoma in situ was 6% and 1.6%, respectively, amongst a cohort of current smokers [167, 168].

The rationale for early detection is the survival of those with intraepithelial neoplastic lesions (stage 0), or early‐stage invasive cancers (Stage 1A – tumour ≤ 2 cm without metastatic spread) of the central airway can be excellent, with 5‐year survival of more than 70% [169, 170, 171]. Micro‐invasive or pre‐invasive lung cancers are still eligible for curative treatment [172].

## 9 Diagnosis of early central lung cancers

Pre‐invasive lesions are not commonly detected as they are often asymptomatic and discovered by chance [163]. The classic screening method for centrally located early lung cancer is sputum cytology. However, this method is limited by low sensitivity [173, 174]. Autofluorescence bronchoscopy (AFB) enables their detection by utilising the spectral differences in fluorescence and absorption properties of normal and dysplastic epithelium [168]. AFB has twice the sensitivity compared to white light bronchoscopy for lesion detection, but its specificity is limited due to false‐positive fluorescence in areas of inflammation or increased epithelial thickness [175]. However, there are data showing abnormal fluorescence and benign histology does impart lung cancer risk which conforms to the recent findings of disordered genomes being present in ‘normal’ airway basal cells [27].

Their mainly silent existence lends to a screening programme. Low‐dose CT scanning misses small central micro‐invasive or pre‐invasive disease [176]. The addition of AFB to low‐dose CT scanning showed success in two small studies [176, 177] but in a larger study of patients deemed high risk for lung cancer, AFB detected too few CT occult cancers (0.15%) to justify the addition of AFB into a lung cancer screening programme [174, 178]. The low detection of CT occult lesions may in part be due to patient selection – in one of the earlier studies, the authors used sputum cytometry as a surrogate for risk to be included in a CT screening trial; 29% of cancers were identified by AFB that were CT occult [177]. Another contributory factor was there were large variations in both the number of biopsies taken across the different trial sites in addition to experience using AFB technology.

To design a screening trial to detect early central lung cancers requires enriching the target population for a greater risk of bronchial premalignant change. Ideally, this would be a less‐invasive approach to a current bronchial airway 280‐gene classifier (necessitating bronchoscopy) that performs well in predicting the presence of pre‐invasive lesions (AUC = 0.92) [179]. The future may consist of AFBs for patients with abnormal cheek or nasal samples akin to colposcopy for patients with abnormal preceding cervical smears. The nasal airway transcriptome is different between smokers and nonsmokers and shares similarity to the transcriptome of the bronchus [180]. Work from the Lung PreCancer Atlas – a multicentre multi‐omic characterisation of premalignant lung lesions – will seek to explore whether genetic changes can be detected noninvasively in the nasal epithelium [181]. The nasal epithelium does not develop squamous cell cancer, which suggests the presence of protective mechanisms, which may pose a challenge in nasal biomarker development.

## 10 Biomarkers to predict pre‐invasive lesion progression

In the largest longitudinal study of treatment naïve pre‐invasive high‐grade lesions, it has been shown that 50% of high‐grade lesions progress to invasive cancer within 2 years and 30% spontaneously regress [158]. In the same study, molecular analyses of the high‐grade biopsy that precedes either progression to invasive disease or regression to lower grade or normal epithelium (the ‘index’ biopsy) has shown that progressive lesions have more mutations compared to regressive lesions, with frequent alterations in known LUSC drivers such as CDKN2a, SOX2 and AKT2 and frequent gains/amplifications at multiple locations on distal 3q. The DNA methylome of regressive index lesions was similar to normal epithelium. The authors devised prediction signatures for progression based on both gene expression (which was able to predict all ‘progressors’ correctly when used on a validation cohort) and DNA methylation (AUC 0.99). The authors also found a considerable number of probes from index lesions had intermediate methylation. The methylation classifer was still able to predict progressive from regressive cases amongst these intermediately methylated regions (AUC = 0.74). Importantly, this methylation heterogeneity that allowed for prediction was a genome‐wide phenomenon and not just related to certain genes functionally implicated in lung cancer development.

Systemic biomarkers for precancerous disease is an area in need of study. The only published study to date investigating ctDNA in pre‐invasive bronchial lesions showed that the amount of ctDNA did not differ amongst patients with pre‐invasive bronchial disease compared to controls [182] but it appears in the literature that no sequencing or methylation analysis has ever been performed.

## 11 Treatment of pre‐invasive squamous cell lung cancer

There is no randomised trial evidence to help decide whether treatment of precancerous lesions is beneficial. Chemo‐preventative trials have had limited success. Inhaled corticosteroids [183]; Sulindac [184], a nonsteroidal anti‐inflammatory drug; Myo‐inositol [185], an inhibitor of the PI3K pathway and Iloprost [186], a synthetic analogue of prostacyclin have all been tested. The latter two did show some anticarcinogenic effects. Spontaneous regression rates as high as 30% make trials difficult. A trial is currently underway to investigate whether Nivolumab (PD‐1 antagonist) can reverse low‐grade dysplasia in pre‐invasive lesions [187]. New drug targets will become available as the molecular biology is better understood. For instance, it has recently been shown that amongst progressive high‐grade lesions, antigen presentation is impaired by both genetic and epigenetic changes; CCL27–CCR10 signalling is altered and the immunomodulator TNFSF9 is downregulated [188].

Surgery is an option but the lesions' central location means most individuals require a lobectomy, carrying appreciable morbidity and mortality that is difficult to justify when there is no guarantee of progression to invasion. Patients also have a field effect indicating high chances of cancer developing elsewhere. Tissue sparing therapies using either electrocautery or photodynamic therapy delivered via a bronchoscope show promise [189, 190].

## 12 Conclusion

The goal of early detection is to facilitate curative treatment in those that will receive benefit from the intervention. Smoking cessation or strategies to prevent smoking initiation are the best means to reduce lung cancer mortality. In the UK, funding for smoking cessation programmes was recently cut by 24% [191].

The last 10 years has seen a trove of potential biomarkers uncovered by having a better understanding of lung cancer biology. Biomarker discovery research is costly and difficult. Large, well‐designed studies are needed, and then, successful discoveries require validation. We hope biomarkers will enter the clinical workspace to supplement risk models and enrich the population for LDCT screening; reduce the false‐positive rates of screen detected nodules and determine screening schedules, and for central early lung cancers to identify patients with aggressive pre‐invasive central lung lesions. The search for biomarkers to detect lung cancers early or in never‐smokers and light smokers has the largest clinical potential but currently seems the furthest away.

## Conflict of interest

SJ: Paid Advisory role for Astra‐Zeneca, Bard1 Bioscience, Achilles Therapeutics, Jansen; received assistance for travel to meetings from Astra Zeneca to American Thoracic Conference 2018 and from Takeda to World Conference Lung Cancer 2019 and is the Investigator Lead on grants from GRAIL Inc, GlaxoSmithKline plc and Owlstone.

## Author contributions

LK wrote the manuscript. RT and SJ reviewed the manuscript and provided expert opinion.

### Peer Review

The peer review history for this article is available at https://publons.com/publon/10.1002/1878‐0261.12864.
